# Complex bud architecture and cell‐specific chemical patterns enable supercooling of Picea abies bud primordia

**DOI:** 10.1111/pce.13078

**Published:** 2017-11-08

**Authors:** Edith Kuprian, Caspar Munkler, Anna Resnyak, Sonja Zimmermann, Tan D. Tuong, Notburga Gierlinger, Thomas Müller, David P. Livingston, Gilbert Neuner

**Affiliations:** ^1^ Institute of Botany, Unit Functional Plant Biology University of Innsbruck Sternwartestr. 15 6020 Innsbruck Austria; ^2^ North Carolina State University and USDA‐ARS 840 Method Road, Unit 3 Raleigh NC USA; ^3^ Department of Material Sciences and Process Engineering, Institute of Wood Science and Technology University of Natural Resources and Life Sciences (BOKU) Vienna Austria; ^4^ Department of Nanobiotechnology, Institute for Biophysics University of Natural Resources and Life Sciences (BOKU) Vienna Austria; ^5^ Institute of Organic Chemistry University of Innsbruck Innrain 80/82 6020 Innsbruck Austria

**Keywords:** cell wall pectins, extraorgan freezing, freeze dehydration, ice nucleation, stem cells

## Abstract

Bud primordia of Picea abies, despite a frozen shoot, stay ice free down to −50 °C by a mechanism termed supercooling whose biophysical and biochemical requirements are poorly understood.

Bud architecture was assessed by 3D—reconstruction, supercooling and freezing patterns by infrared video thermography, freeze dehydration and extraorgan freezing by water potential measurements, and cell‐specific chemical patterns by Raman microscopy and mass spectrometry imaging.

A bowl‐like ice barrier tissue insulates primordia from entrance by intrinsic ice. Water repellent and densely packed bud scales prevent extrinsic ice penetration. At −18 °C, break‐down of supercooling was triggered by intrinsic ice nucleators whereas the ice barrier remained active. Temperature‐dependent freeze dehydration (−0.1 MPa K^−1^) caused accumulation of extraorgan ice masses that by rupture of the shoot, pith tissue are accommodated in large voids. The barrier tissue has exceptionally pectin‐rich cell walls and intercellular spaces, and the cell lumina were lined or filled with proteins, especially near the primordium. Primordial cells close to the barrier accumulate di, tri and tetrasaccharides.

Bud architecture efficiently prevents ice penetration, but ice nucleators become active inside the primordium below a temperature threshold. Biochemical patterns indicate a complex cellular interplay enabling supercooling and the necessity for cell‐specific biochemical analysis.

## Abstract 2

Mechanisms of freeze avoidance and supercooling are poorly understood, although they are widely present frost survival mechanisms in tissues of woody plants. Despite a frozen shoot, vegetative bud primordia of Picea abies are able to supercool down to −50 °C. This is achieved by a sophisticated bud architecture, which insulates the bud primordium from external and internal ice penetration. The ice barrier tissue and adjacent cell layers show a highly differentiated chemical composition facilitating supercooling of Picea abies bud primodia.

## 1 INTRODUCTION

Overwintering bud primordia of Picea abies survive freezing temperatures by supercooling (Buchner & Neuner, 2009; Pukacki, 1987; Räisänen, Repo, Rikala, & Lehto, 2006). Supercooling involves extraorgan freezing and can generally be found as a frost survival mechanism in vegetative bud primordia of conifers within the genera *Abies*, *Larix*, and *Picea*, but not in *Pinus* (Sakai & Larcher, 1987).

Supercooling bud primordia are less frost resistant comparatively to other organs (Sakai & Larcher, 1987; Wisniewski, Neuner, & Gusta, 2015). Those are usually killed around −40 °C, but ultimately at −50 °C during midwinter (Beuker, Valtonen, & Repo, 1998). During freezing, primordia become successively dehydrated. The extracted water freezes extraorgan. A certain amount of supercooled liquid water remains in the cells of the bud primordia. However, when supercooling capacity is exceeded, the cells freeze internally and are killed.

At slow cooling rates, ice nucleation is initiated extracellularly, in ice tolerant plant tissues (Hacker & Neuner, 2007). Extracellular ice formed in the shoot of P. abies between −8.7 °C and −10 °C (Buchner & Neuner, 2009; Pukacki, 1987). In plants, ice nucleation can be intrinsic or extrinsic (Pearce, 2001), but once initiated, it spreads at rates up to 27 cm s^−1^ throughout all plant parts that are colder than 0 °C (Hacker, Ladinig, Wagner, & Neuner, 2011; Hacker & Neuner, 2007, 2008; Hacker, Spindelböck, & Neuner, 2008; Neuner & Hacker, 2012). Therefore, an ice barrier between the frozen shoot and the bud primordium that restricts the spread of ice into the ice susceptible primordium is necessary. Additionally, the bud primordium must be protected against extrinsic nucleation from the bud surface by an external ice barrier. Structural requirements may be met by a peculiar water or ice proof bud architecture, where the bud scales and cuticle may play an important role (Quamme, Su, & Veto, 1995).

Despite an already frozen shoot, lethal ice formation in the bud primordium of P. abies is recorded between −16 °C and −50°C in midwinter (Beuker et al., 1998; Neuner & Bannister, 1995; Pukacki, 1987). However, the overall freezing pattern around bud primordia is not known. Ice nucleation of the bud primordium could occur principally in three ways, either by extrinsic nucleation, by breakdown of the ice barrier and subsequent spreading of ice from the frozen shoot into the primordium, or by an independent ice nucleation event inside the primordial cells.

To understand freezing patterns and the movement of water and ice in shoots and buds of P. abies, knowledge of the architecture of the overall bud, the connecting tissues, and the subtending shoot is indispensable as recently shown for Calluna vulgaris (Kuprian et al., 2017). Information about bud anatomy in conifers is scarce (Cesar & Bornman, 1996; Curtis & Popham, 1972; Lewis & Dowding, 1924). However, in supercooling buds of *Picea*, *Pseudotsuga*, *Larix*, and *Abies,* a so‐called crown tissue separates the shoot from the bud tissues (Lewis & Dowding, 1924), whereas buds of the genus *Pinus* that do not supercool lack this crown tissue (Sakai, 1978). The crown tissue in buds of P. abies (Cesar & Bornman, 1996; Curtis & Popham, 1972; Lewis & Dowding, 1924; Sakai, 1978) might serve as a structural ice barrier and aid supercooling. Cytological and histological investigations of bud architecture of P. abies should provide clear evidence for the fundamental structural properties that can restrict the intrinsic spread of ice.

Extraorgan freezing theory suggests that freezing of apoplasmic water in the stem and subtending bud scales, whereas a supercooled primordium, with decreasing temperature, produces a water potential gradient and a consequent migration of primordial water to the ice below (Ishikawa & Sakai, 1981). However, the extent and the means by which these occur are not well understood. The boundary tissue must function as an ice barrier but must likewise allow water movement to the already formed ice masses. Ice nucleation and potential resublimation in the supercooled primordium must also be impeded.

Supercooling in overwintering vegetative buds has been detected in some conifers, but until now, only in reproductive buds of angiosperms. For these species, features of the ice barrier tissue include the absence of an intact xylem connection, that is, provascular strands (*Rhododendron spec*.: Ishikawa & Sakai, 1981), small tightly packed cells with thick cell walls that are devoid of vacuoles and intercellular spaces (e.g., Prunus persica: Ashworth, 1984; Quamme et al., 1995; *Vitis spec*.: Jones, McKersie, & Paroschy, 2000), impregnation of cell walls with phenolic compounds (suberin and lignin in *Rhododendron* species: Chalker‐Scott, 1992), accumulation of unesterified pectins (P. persica: Wisniewski & Davis, 1995), and regions of dry tissue (Rhododendron japonicum: Ishikawa & Sakai, 1981 *,* P. persica: Quamme, 1978). The ability to supercool may depend on cell wall composition. Particularly, effects on cell wall porosity may play a role because water in small diameter pores freezes at lower temperatures than water in larger pores (Ashworth & Abeles, 1984; Zwiazek, Renault, Croser, Hansen, & Beck, 2001).

The peculiar structural features and the chemical composition of cell walls in the crown tissue of P. abies and its functional role as an ice barrier have not been researched. Therefore, using light and Raman microscopy, as well as mass spectrometry imaging (MSI) and infrared video thermography we examined biochemical and biophysical characteristics that enable the bud primordium to remain ice‐free and supercooled during freezing.

Using this comprehensive set of methods, we are going to answer the following questions: (a) What anatomical requirements must be met to avoid extrinsic and intrinsic ice nucleation to keep the bud primordium in a supercooled state? (b) Do the ice barriers break down below a certain low freezing temperature or are separate ice nucleation events in the primordium observed? (c) Is temperature‐dependent freeze dehydration observed and to what extent does it contribute to supercooling of bud primordia? (d) Does a peculiar chemical composition of the cell walls and cellular content in the ice barrier tissue and the primordium itself facilitate supercooling?

## 2 MATERIAL AND METHODS

### 2.1 Plant material

Twigs with buds of P. abies (L.) H. Karst. were collected in the Botanical Garden of the University of Innsbruck (613 m; 47°16′4″N, 11°22′43″E) from five randomly chosen individuals out of 60 potted trees cultivated outdoors under natural field conditions. Investigations were conducted on terminal and lateral vegetative buds of P. abies when the overall bud had at least 2 mm in size.

### 2.2 Differential thermal analysis

After sampling, differential thermal analysis (DTA) measurements were conducted according to Burke, Gusta, Quamme, Weiser, and Li (1976). On 2–3 cm long twig pieces, supernumerous buds were removed to ensure an unambiguous detection of freezing exotherms from the investigated bud. Temperatures were measured with copper‐constantan thermocouples (T‐type, fine wire, and solder junction diameter 0.12 mm), fixed at the outer bud scales. Appropriate pieces of thermally conducting, self‐adhesive pads (Laird Technologies, Earth City, Missouri, USA) were wrapped around the bud and the fixed thermocouple before being surrounded with aluminium foil and inserted into wells with various diameters from 6 to 10 mm that had been drilled into aluminium cylinders (diameter 10 cm, height 10 cm).

Dead reference samples that had been dried at +80 °C for 72 h, were frozen in the same way as living bud samples. The aluminium solids with samples were put into the cooling compartment of a temperature‐controlled commercial freezer (Profiline Taurus, National Lab GmbH, Mölln, Germany). Controlled cooling (software developed by Othmar Buchner in LabView 2012) started at +5°C, and then the freezer temperature was lowered at 3 K.h^−1^ to below the frost killing temperature of the buds.

The temperature was monitored using thermocouples connected to a multiplexer (AM 416, Campbell Scientific, Logan, UT, USA) and recorded with a data logger (CR10 Campbell Scientific) at an interval of 10 s. DTA values were obtained by subtracting the temperature of the reference twig from the temperature of the sample twig. The high temperature exotherm (HTE) and the low temperature exotherm (LTE) were determined graphically from the DTA plot as the starting temperature of the respective freezing exotherm.

### 2.3 Frost resistance of bud primordia

Frost resistance of vegetative buds of P. abies was determined by exposing shoots bearing buds to a set of different freezing temperatures. At least 10 buds randomly chosen from twigs were taken at 2‐week intervals from five individual potted trees. These twigs were placed on wet paper towels inside sealed plastic bags. A set of twigs was kept in a cooling chamber at +4 °C to +6 °C as an untreated control. Bud samples that were meant to be frozen were put inside sealable plastic bags into eight freezers (Liebherr, Lienz, Austria) and exposed to different target temperatures (5 K intervals) between −5 °C and −50 °C. From +2 °C cooling proceeded at a rate of 3 K h^−1^, down to target temperatures, which were held for 4 h. Thawing took place at a rate of 3 K.h^−1^.

Viability of bud primordia was once assessed by triphenyltetrazolium chloride (TTC) staining and in parallel by measuring chlorophyll fluorescence of bud primordia, using a MINI PAM (Walz, Effeltrich, Germany). Until now, chlorophyll fluorescence has been used as a viability assay on leaves (Bannister et al., 2005; Neuner & Buchner, 1999) but not for bud primordia. As no significant differences between the results of these two methods were observed (data not shown), we further on employed the chlorophyll fluorescence technique for viability assessment on bud primordia being more objective, fully quantitative and less time consuming.

The Fv/Fm values were converted into percentage damage values by setting the untreated control to 0% and frost killed samples to 100%. The percent frost damage was then plotted against the target temperature and a logistic function (Boltzmann function) was fitted to the data using OriginPro 7G (OriginLab Corporation, Northhampton, USA). From the parameters of the logistic function, the LT_50_‐value, that is, the temperature at 50% frost damage, was determined. Mean values and standard deviation of these LT_50_‐values were calculated for each sampling date.

### 2.4 Infrared differential thermal analysis

Ice nucleation, propagation, and supercooling within and around vegetative buds of P. abies were monitored with a digital infrared camera (T650SC, FLIR Systems, Danderyd, Sweden). This infrared camera has a thermal resolution of 0.2 mK. We used a close‐up lens (magnifying factor: 1.5×, working distance: 46 mm) to obtain a spatial resolution of 25 μm. Infrared images were recorded at an interval of 10 frames per second. Subsequent processing of the images by infrared differential thermal analysis (IDTA; Hacker & Neuner, 2008; Neuner & Kuprian, 2014) was conducted with FLIR ResearchIR Max (version 4.20.2.74, FLIR Systems). Image overlay was done with After Effects (Adobe Systems Inc., San Jose, USA).

A longitudinal section of the terminal part of a twig of P. abies, bearing three vegetative buds, was mounted on a small lift table by double‐sided adhesive tape. In the image field of the camera (16 mm × 12 mm), the three buds were arranged in a focal plane. Additionally, six thermocouples were mounted close to the buds. Thermocouple output was recorded every 10 s by a data logger (CR10X, Campbell Scientific).

### 2.5 Histological analysis, sectioning, and staining

For histological analysis of the vegetative buds of P. abies, twigs were detached from 10 randomly selected trees in December 2014 and January 2016. From these twigs, about 20 buds and subtending shoots were selected and fixed in methanol‐formaldehyde‐acetic acid‐alcohol (methanol 45%, formaldehyde 10%, glacial acetic acid 5% and distilled water 40%; Livingston, Tuong, Haigler, Avci, & Tallury, 2009) that was at a similar temperature as the buds. This fixes the tissue in its frozen state at the same time the ice is being melted. The freeze‐fixation protocol was adapted from a technique used to image ice in rodent hearts (MacKenzie, Kuster, & Luyet, 1975). After warming to room temperature, samples were shipped to Raleigh (NC, USA), where they were further processed in the Livingston laboratory. Samples were additionally treated with methanol‐formaldehyde‐acetic acid‐alcohol and then taken through an ethanol dehydration series and embedded in paraffin using a microwave‐assisted protocol (Livingston et al., 2009).

Paraffin embedded tissues were sectioned with a rotary microtome (Leica, Model RM2255, Wetzlar, Germany) at 20 μm and triple stained with safranin, fast green, and orange G (Livingston et al., 2009). Safranin stains lignified, cutinized, and suberized structures, whereas fast green stains cellulose walls and cytoplasm (Johansen, 1940). Sections were photographed with a Canon Rebel T3i (Canon Inc, Taipai, Taiwan) mounted on a Nikon Eclipse 50i light microscope (Nikon Corporation, Tokyo, Japan) at 20× and 100× magnification. A 10 mm, 100 part photo‐reticle was used to calibrate measurements in all images.

### 2.6 3D reconstruction

To visualize frozen buds in three dimensions, 341 sequential images of freeze‐fixed and stained sections were imported into After Effects (Adobe Systems Inc.). Images were aligned manually and background color was removed using color‐clearing subroutines within After Effects as described (Livingston et al., 2010). Voids were considered to be a result of ice formation when at least three to five sequential images had similar tissue or cell separation at the same anatomical location. Sections from frozen plants were also compared to unfrozen controls and only voids that were not present in sections from unfrozen plants were considered to be caused by ice formation. In addition to voids caused by ice formation, resin ducts within the buds were also identified (Videos S1 and S2). Voids were identified and color coded using the Roto‐Brush tool within After Effects. The animated videos shown in the Supporting information were created as described by Livingston and Tuong (2014).

### 2.7 Total water potential

Total water potential of the bud primordia of detached vegetative buds of P. abies were determined using eight C‐52 chambers (Wescor Inc., Logan, UT, USA). The chambers were connected to a PSYPRO water potential system (Wescor Inc.). The PSYPRO system measures water potentials on the basis of psychrometric recordings. The C‐52 chambers were used with sample holders with a diameter of 7 mm and a depth of 2.5 mm.

The C‐52 chambers were calibrated with NaCl solutions (OPTI‐MOLE™ Osmolality Standards, manufactured for Wescor, Inc.) with concentrations of 100, 290, and 1,000 mmol kg^−1^. These standards were applied to 6 mm diameter filter paper discs (Whatman, Schleicher & Schuell, type 597, pore size 4–7 μm).

Water potential was measured on isolated bud primordia, which were dissected from the shoot tissues with a razor blade. To ensure that only primordial cells were measured, all other tissues below the primordium were removed. This was done by cutting off layer after layer from the direction of the subtending shoot to the primordium, until the nearly transparent cells of the crown tissue got visible. Bud scales were removed by a gentle twisting and squeezing motion.

### 2.8 Raman spectroscopy

Raman imaging reveals the chemical composition of plant cell walls in context with the microstructures with a spatial resolution smaller than 0.5 μm in a nondestructive manner (Gierlinger, Keplinger, & Harrington, 2012). Buds were collected in December 2014 and January 2015, put in a sealable plastic bag with wet filter paper, and stored at +5 °C overnight. Sample preparation, acquisition of hyperspectral images, and the final spectra and image analysis were done in the Gierlinger laboratory (University of Natural Resources and Life Sciences, Vienna). Preliminary various sample preparation methods and instrumental acquisition parameters were tested to get the best insights into the topo‐chemistry of the buds in vivo. Finally, best results have been achieved using 200 μm thick sections, cut with a rotary microtome (RM2235, Leica Biosystems Nussloch Gmbh, Germany) and glued (Loctite superglue) onto a metal plate, which was placed on a magnet within a custom built sample device to measure under water. Raman spectra from the native cross sections were acquired as maps of different dimensions using a confocal Raman microscope (alpha300RA, WITec GmbH, Germany) with a 63× water immersion objective (NA = 1) (Carl Zeiss, Germany). The 785 nm laser line (diode laser, CrystaLaser, Reno, NV, USA) turned out to be best suited, as sample fluorescence had been reduced. A good signal to noise ratio was obtained by using an optimized blazed grating (600 g mm^−1^, UHTS spectrometer, WITec Germany) and a deep depletion charge‐coupled device camera (ANDOR, DU401A BR‐DD) to record the Stokes scattering. Cosmic ray removal and the average spectra, as well as the Raman images, were calculated using the WITec Project software. Different samples, positions, and scan sizes were measured, and the biggest and longest scan, representing the most comprehensive one, is shown in this paper. Within the selected area (Figure 6, rectangle, 50 × 250 μm), 78,125 spectra (125 × 625 pixel) were acquired with an integration time of 1.2 s and a laser power of 100 mW. Although many bands are overlapping, integration over some characteristic bands allowed us to follow overall chemical composition as well as differences within the investigated region.

### 2.9 Mass spectrometry imaging

From 10 collected twigs, single buds were randomly chosen and longitudinally cut (about 20‐μm thick) by the use of a microtome (GSL1‐microtome; Gärtner, Lucchinetti, & Schweingruber, 2013). The most suitable, freshly cut bud sections were fixed on top of a stainless steel matrix‐assisted laser desorption/ionization (MALDI)‐target using a double‐sided carbon tape. Eleven milligrams of solid 2,5‐dihydroxybenzoic acid were dissolved in 1 ml of methanol. Methanol (HPLC grade) was bought from Fisher Scientific (Louborough, UK), 2,5‐dihydroxybenzoic acid from Acros Organics (Geel, Belgium) and the conductive double‐sided carbon tape from Electron Microscopy Sciences (Hatfield, USA). An in‐house built sprayer (Vergeiner, Schafferer, Haas, & Müller, 2014) was used to apply 0.5 ml of the matrix solution on a sample area of about 3 cm^2^ (flow rate was 200 μL min^−1^; N_2_ was used as nebulizing gas at 6 bar; and distance from sample to sprayer tip was kept at approx. 7 cm). MALDI‐time‐of‐flight (TOF) MSI was performed using a Bruker Ultraflex MALDI TOF mass spectrometer equipped with a smartbeam Nd:YAG‐laser with an effective spot size of 35 μm; the laser power was adjusted to 80% (manufacturer's arbitrary units). In positive ion reflectron mode, the sample was rastered at a lateral resolution of 100 × 100 μm and 100 MS spectra in the mass range of m/z 100 to m/z 2,000 were averaged at every single spot. Data were analyzed using FlexImaging 2.1 software (Bruker Daltonics). The mass filter ranges for the generation of ion images were set to ± 0.025% of the monoisotopic molecular ions' m/z value.

### 2.10 Statistical analysis

Mean values of ice nucleation temperatures (of the primordium of P. abies [LTE]) and frost resistance of the primordia for each season (*n* = 9 in summer, *n* = 19 in winter) were calculated (see Figure 3). The supercooling ability of bud primordia depending on different treatments was tested on 44 controls, 24 damaged (“cut”), and 24 infiltrated buds (see Figure 4). Differences between mean values with homoscedasticity were tested with a one‐way analysis of variance (anova) and a subsequent Duncan post hoc‐test. In case of a negative Levene‐test, the Mann–Whitney *U*‐test was used as a nonparametric post hoc‐test (spss Statistics, IBM Corp.,Version 21.0., Armonk, NY). All analyses were conducted at a significance level of *p* ≤ .05.

## 3 RESULTS

### 3.1 Structural features of overwintering vegetative P. abies buds

**Figure 1:**
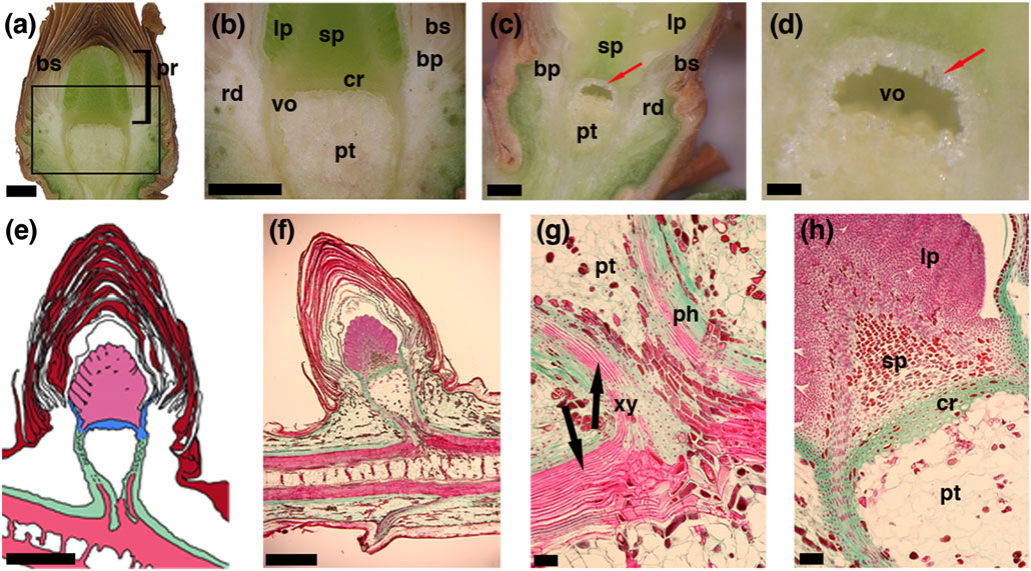
Images and a schematic drawing of longitudinal sections through vegetative buds of Picea abies. (a,b) Digital images of a longitudinal section through an unfrozen (April 2016) and (c,d altered from [Neuner & Hacker, 2012]) frozen bud (June 2008). Red arrows (c,d) indicate location of ice crystals accumulated by translocated ice formation (extraorgan freezing). (e) Schematic drawing of a longitudinal section through the bud, where the ice barrier tissue (blue) formed by the crown and the inner bark tissue (in 3D a bowl‐shaped structure) encapsulates the primordium (light pink). (f,g,h) Microscopic images of a longitudinal semithin section of a vegetative bud of Picea abies before a seasonal freezing event after triple staining with safranin, orange G, and fast green. (g) Enlargement of the lower box shown in F. (h) Enlargement of the upper box in F. The phloem is colored green, the xylem pink, and the bud scales are colored red‐brown. Horizontal bars indicate 1 mm in a, b, c, e and f, 0.25 mm in d, and 0.1 mm in g and h. Abbreviations: bs = densely packed bud scales; pr = primordium; sp = shoot primordium; lp = leaf primordia; pt = pith tissue; rd = resin ducts; bp = basal part of the bud scales, cr = crown; vo = void between the crown and the pith tissue; ph = phloem; xy = xylem

The vegetative buds of P. abies consist of various highly differentiated tissues (Figure 1a,b). The chlorophyll containing primordium inside the vegetative buds of P. abies is covered by numerous densely packed bud scales and consists of a shoot primordium and many attached leaf primordia (3D reconstruction: Video S1). The shoot primordium is separated from the mature shoot tissues by a distinct tissue just below it, called the crown (Lewis & Dowding, 1924), which is about 0.21‐mm thick (see Video S1). The crown tissue consists of 3–8 cell layers with thicker cell walls than surrounding cells and lacks intercellular spaces. In the frozen state, ice crystals accumulated and formed a void (Figure 1c,d; see Video S1 blue colored space) below the crown tissue, whereas the primordium above this supercooled and remained free of ice. The ice void, formed by accumulation of ice masses during freezing, measured about 0.87 mm in height. These ice masses destroyed the thin walled cells of the pith parenchyma tissue. Unfrozen, control buds did not contain voids between cells in any tissue (see Figure 1a,b).

Longitudinal stained sections (Figure 1f–h) confirmed that the primordium is separated from the tissue of the rest of the bud. Densely packed thick‐walled cells of the crown tissue form what appears to be a barrier. This tissue extends laterally upwards forming a bowl‐like structure, wherein the primordium is located. The xylem was not yet developed into the primordium as the differentiated vascular tissue ends about 1 mm below the crown tissue (see Figure 1f–h). The bud primordium was covered by numerous densely packed bud scales (Video S2) that form air cushions, encapsulate and protect the primordial tissue from the surrounding environment.

### 3.2 Supercooling of the bud primordium

**Figure 2:**
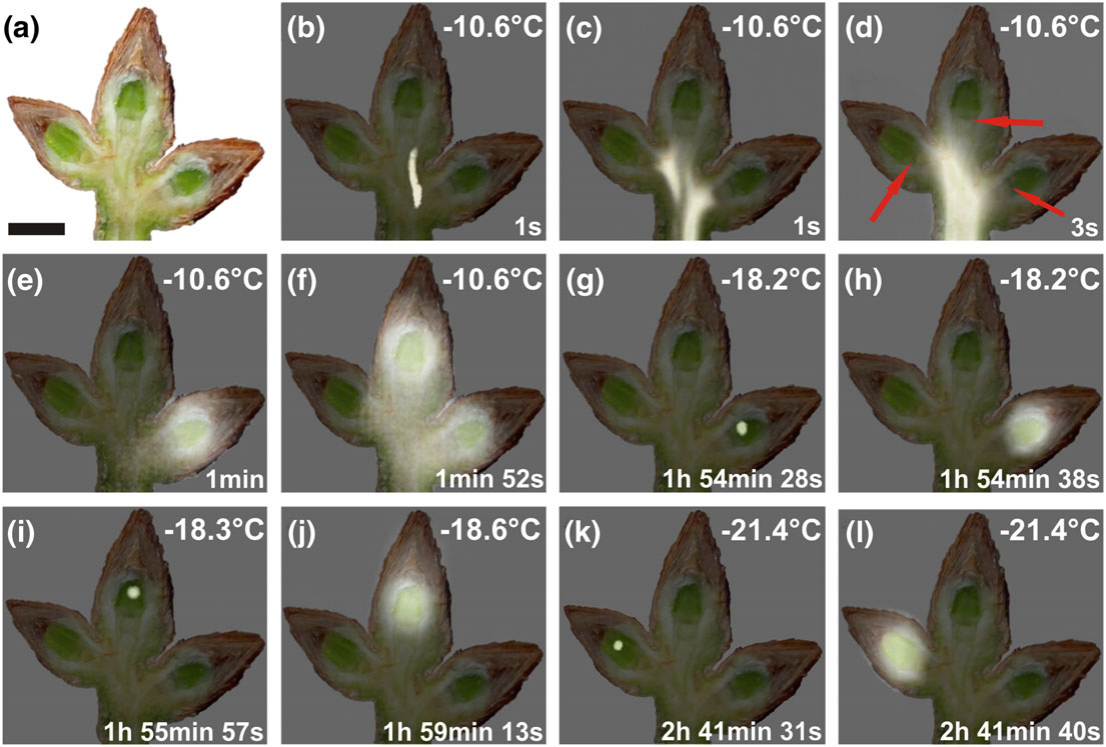
Digital image and infrared differential thermal analysis images of buds of Picea abies during controlled freezing in October 2015. (a) Digital image of a longitudinally sectioned shoot of Picea abies bearing three vegetative buds taken immediately before the onset of a freezing treatment. (b–l) Sequence of images of an overlay of the digital image and infrared differential thermal analysis images during controlled freezing. Whitening indicates freezing events, whereas unfrozen areas remain unchanged. Ice nucleated at −10.6 °C in the shoot and propagated throughout the whole shoot (b–d). The structural ice barriers are clearly detectable below each bud primordium (d, red arrows). Further, nonlethal freezing events followed approximately 1 min after the initial rapid spread of ice throughout the shoot and seem to originate from freezing of extracellular water in the bark tissue and of the base of the bud scales aside and in the background of each bud primordium (e,f). All bud primordia remained supercooled and froze individually and at significantly lower temperatures between −18.2 °C and −21.4 °C, about 1.9 h and 2.7 h, respectively, later than the initial ice nucleation in the shoot. Actual temperatures are indicated in the top right corner of each image. The time span (in hours, minutes, and seconds) after initial ice nucleation in the shoot is indicated at the bottom right corner. Black bar = 0.5 cm

IDTA clearly demonstrates the supercooling ability of the bud primordia of P. abies (Figure 2). Under the experimental conditions the primordia remained in a supercooled state for up to 2.7 h. After nonlethal initial ice nucleation (HTE) in the shoot tissue at −10.6 °C, ice rapidly propagated unhindered throughout the whole shoot (Figure 2b–d). However, below each bud, the ice wave stopped approximately where the differentiated vascular tissue ended (see Figure 1g, below the crown tissue). This suggests that the crown tissue serves as an ice barrier. Immediately after this initial spread of ice, freezing events in the periphery of the buds were detected. By image overlay, these second freezing events at the end of HTE could be localized to originate from the basal parts of the bud scales and the subtending tissue that encases the bowl shaped crown tissue (Figure 2e,f). Additionally, they were not lethal as frost damage to bud primordia was recorded at much lower freezing temperatures (mean LT_50_–24.2 °C). Under the experimental conditions, all three bud primordia supercooled and remained ice‐free down to at least −18.2 °C. After separate initial ice nucleation events (LTE; Figure 2g,i,k) inside each primordium at −18.2°C, −18.3°C and −21.4°C, they froze individually (Figure 2h,j,l), killing the entire primordium (verified in regrowth experiments, data not shown). The site of ice nucleation inside the primordial tissues was in all cases distinctly distant from the crown tissue (at mean 2 mm). This suggests that the ice barrier function of the crown tissue could be maintained throughout but that ice nucleators got active in the supercooled primordial cells.

**Figure 3:**
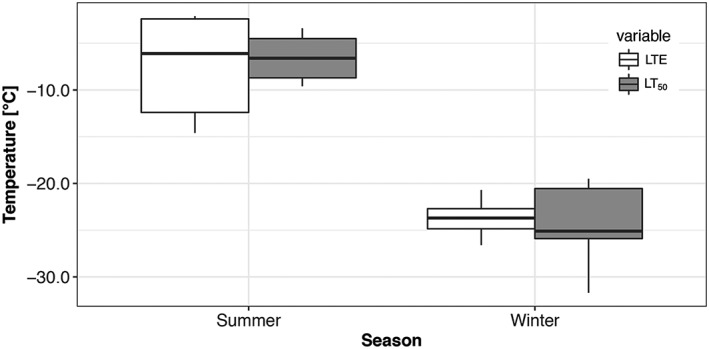
Low temperature exotherms (LTEs) and frost resistance of supercooled primordia of vegetative buds of Picea abies (*n* = 9 in summer, *n* = 19 in winter) recorded during controlled freezing treatments in late August 2013 and in December/January 2013/2014. LTEs in summer were recorded at significantly higher temperatures (mean −7.1°C ± 1.8 SE) than in winter (mean −23.8 °C ± 0.4 SE). Frost resistance (LT_50_) is significantly higher in winter (mean −24.2 °C ± 0.8 SE) than in summer (mean −6.6°C ± 0.8 SE). The box plots indicate the median (= second quartile; line inside the box) and extend from the first to the third quartile. The whiskers show at most the 1.5‐fold interquartile range

The supercooling ability (LTE) and the frost resistance of bud primordia of P. abies increased significantly from the end of summer till midwinter (Figure 3). Although mean LTE was observed at −7.1 ± 1.8 °C in late August 2013, LTE decreased under natural hardening conditions to a value of −23.8 ± 0.4°C in December 2013/January 2014. Similarly, frost resistance of bud primordia increased significantly from −6.6 ± 0.8°C to −24.2 ± 0.8°C.

### 3.3 Protection from extrinsic ice nucleation

**Figure 4:**
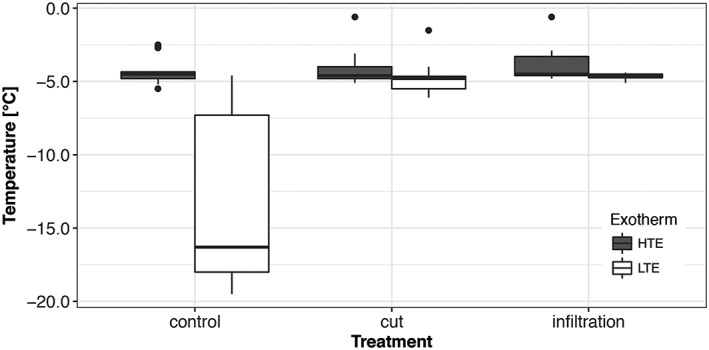
LTEs of supercooled primordia of vegetative buds of Picea abies (*n* = 44 “control,” *n* = 24 “cut,” and n = 24 “infiltration”) recorded during three controlled freezing treatments in April/May 2014. Half of the buds were either infiltrated with water by the use of a vacuum pump or the tip of the bud scales was cut off with a razor blade without damaging the primordium. By these treatments, supercooling ability of the bud primordia was completely lost. The box plots indicate the median (= second quartile; line inside the box) and extend from the first to the third quartile. The whiskers show at maximum the 1.5‐fold interquartile range. Outliers are shown as black dots. LTE = low temperature exotherms; HTE = Low temperature exotherms

Mechanical damage or substitution of air cushions between bud scales and the primordium surface by vacuum infiltration with water, caused an immediate loss of supercooling ability (Figure 4). This demonstrates that an intact encapsulation by bud scales is essential to avoid extrinsic ice nucleation. DTA results on infiltrated buds (Figure 4) combined with the histological structure of the bud (Figure 1e) indicate that the ice wave may in this case also pass from the bark tissue surrounding the bowl shaped crown into the water filled spaces between bud scales and penetrate from there unhindered into the primordium.

### 3.4 Freeze dehydration of bud primordia across the crown tissue and extraorgan freezing

**Figure 5:**
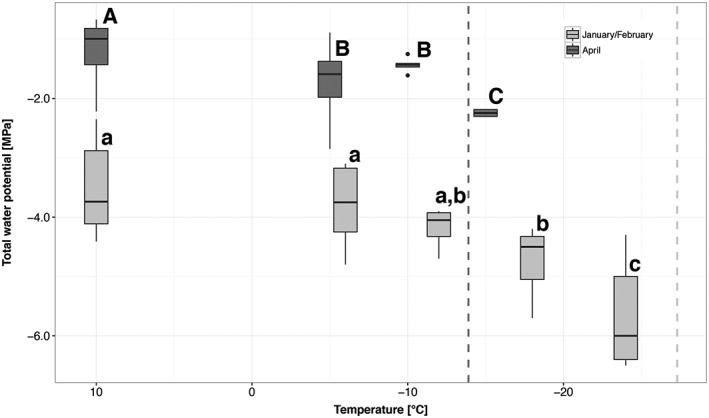
Successive freeze dehydration of primordia of vegetative buds of Picea abies during a controlled freezing treatment in April 2013 (dark grey boxes) and January/February 2015 (light grey boxes). Initial frost damage (LT_i_) is indicated by a dashed line and was −27.3 °C in winter 2015 (dark grey) and −13.9 °C in April 2013 (light grey). When the buds reached a temperature of −15.0 °C and −18.0 °C in April 2013 and January 2015, respectively, the total water potential of primordia was significantly more negative. Significant differences between the total water potential of primordia at different temperatures are indicated by capital letters (April 2013) and lower case letters (January/February 2015). The box plots indicate the median (= second quartile; line inside the box) and extend from the first to the third quartile. The whiskers show at maximum the 1.5‐fold interquartile range. Outliers are shown as black dots

When bud primordia were excised from frozen shoots in the supercooled state during a controlled freezing treatment (−3 K h^−1^), total water potential values indicated successive moderate freeze dehydration (Figure 5). Total water potential of supercooled bud primordia became more negative with decreasing temperature and reached a mean value of −5.7 ± 0.4 MPa at −24 °C in January/February 2015 and −2.2 ± 0.1 MPa at −15°C in April 2013. This equals a freeze dehydration rate of −0.09 MPa K^−1^ and ‐0.06 MPa K^−1^, respectively. Withdrawn water across the crown tissue formed large ice masses in the pith tissue of the shoot (extraorgan ice; see Figure 1d). Unfortunately, gradual freezing of the translocated water during cooling could not be tracked by IDTA or DTA.

### 3.5 Chemical patterns

The ice barrier tissue stained primarily green, indicating cellulose and functional cytoplasm (Ruzin, 1999). Interspersed with and surrounding this tissue were red‐staining cells indicating lignified or cutinized structures (Ruzin, 1999).

**Figure 6:**
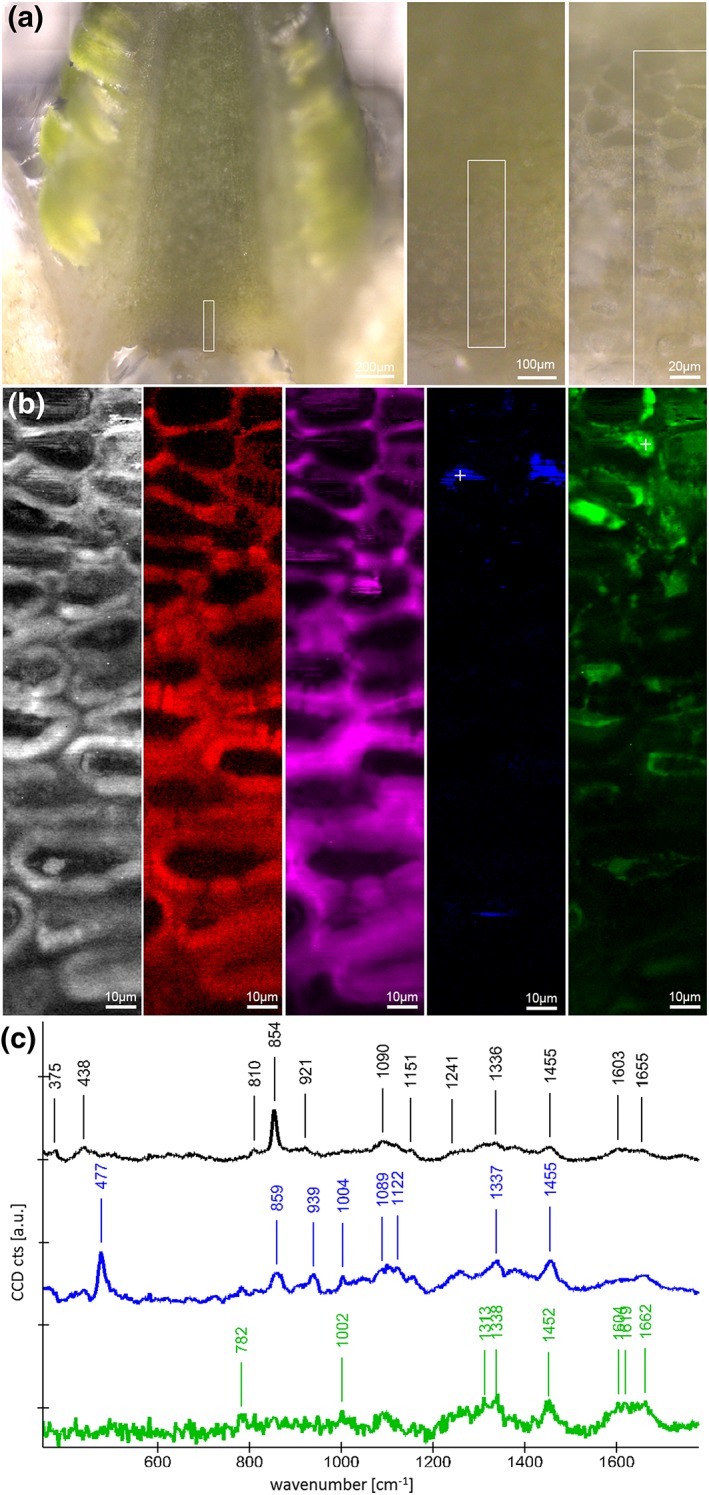
Raman imaging on a microcut winter bud of Picea abies: (a) The region of interest was restricted to the crown tissue as marked in the light microscopic overview images. After spectra acquisition, Raman images (b) were calculated by integrating from 1,056 to 1,137 cm^−1^ (black and white, all structures), 370 to 385 cm^−1^ (red, cellulose), 838 to 868 cm^−1^ (pink, pectin), 462 to 500 cm^−1^ (blue, starch), and 1,523 to 1,700 cm^−1^ (green, protein). (c) Extracted Raman spectra reveal details on the molecular structure of the whole investigated region (black spectrum) and the starch (blue spectrum) and protein (green spectrum) rich deposits. White crosses in the Raman images mark the position of the extracted starch and protein spectrum. CCD = charge‐coupled device

To identify the in vivo chemical composition of tissue within the buds of P. abies by Raman imaging, it was necessary to section the inner bud without any chemical transformation (e.g., fixation) and dehydration (as necessary for most common histological procedures). Raman images elucidate the cell wall as well as deposits within the cell lumina, with a diffraction limited spatial resolution (*r* = 0.6xλ/NA, in the shown scan r = 0.6x785nm 1^−1^ ~ 500 nm, Figure 6b). The grey‐scale image shows all organic structures by integrating a broad region from 1,056 to 1,137 cm^−1^, assigned mainly to C–C and C–O‐stretching vibrations of carbohydrates (Wiley & Atalla, 1987). When we integrated selectively for cellulose using the band at 375 cm^−1^, only the cell wall was highlighted (Figure 6b, red image). By focusing on the characteristic pectin band at 854 cm^−1^ (Synytsya, Copikova, Matejka, & Machovic, 2003), pectin distribution was followed (Figure 6b, pink image). Although cellulose is restricted to the cell wall, pectin is not only found in the cell wall, but to a greater extent as an adhesive in the intercellular spaces between the cells. An average spectrum extracted from the whole scan of the ice barrier tissue shows clearly the high concentration of pectin (band at 854 cm^−1^) and to less extent cellulose signals (band at 1090 cm^−1^ and 375 cm^−1^) (Figure 6c, black spectrum). The thick walls of the crown and the thinner walls towards the primordium were therefore confirmed to be pectin‐rich cellulosic cell walls. Furthermore, contributions from proteins were suggested by the 1,655 cm^−1^ band (amide I stretching vibration of C=O, (Rygula et al., 2013). Integrating the starch specific band at 477 cm^−1^ (Liu et al., 2015) indicated that in a few cells near the primordium starch accumulates (Figure 6b, blue image). Extracting a single spectrum from one of those regions (white cross) confirmed the presence of starch with the strong 477 cm^−1^ band (assigned to C–C–C bending and C–O torsion) as well as strong bands at 859, 939, 1,089, 1,122, 1,337, and 1,455 cm^−1^ (Figure 6c, blue spectrum). Comparing band positions of the bud sample of P. abies with positions reported for crystalline and amorphous starch, points to a more amorphous state of the starch within the cells (Liu et al., 2015) whereas the band at 1,004 cm^−1^ indicates the presence of phenylalanine (proteins). Proteins, as visualized by integrating the Amide I region from 1,523 to 1,700 cm^−1^ (Figure 6b, green image), lined the cell walls towards the lumen and nearly filled some cells, especially towards the primordium. An extracted (Figure 6b, white cross in the green image) single spectrum shows typical protein bands (Figure 6c, green spectrum) and confirms that proteins are partly filling up cells in the investigated region (Figure 6b, green image).

**Figure 7:**
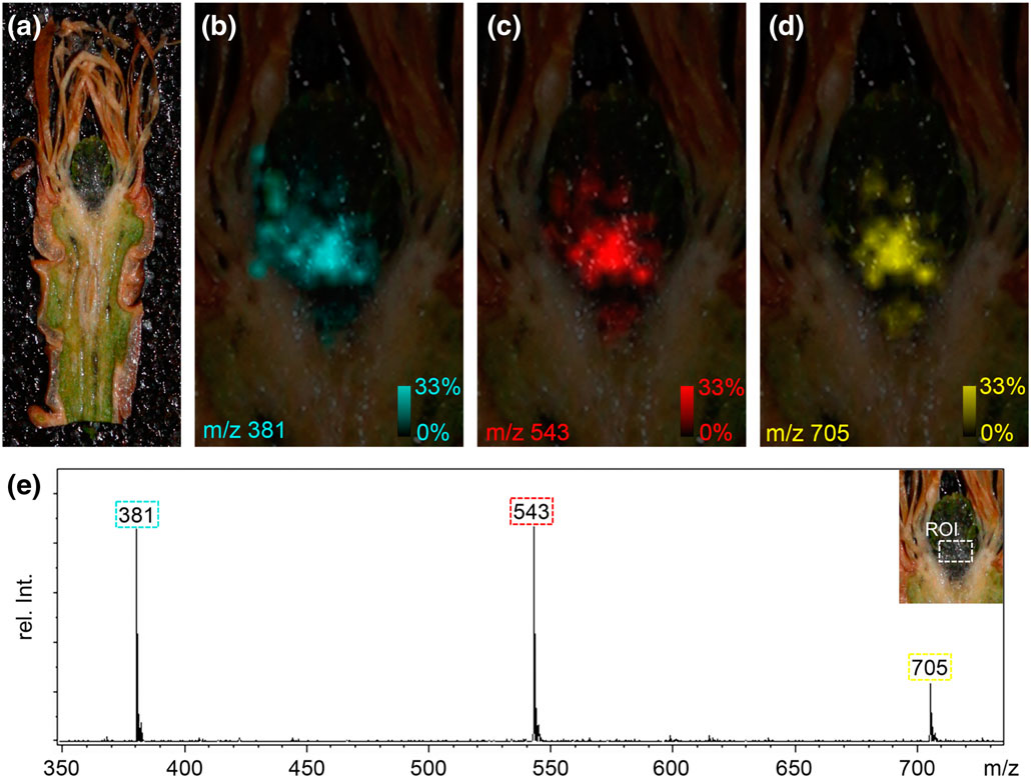
Matrix‐assisted laser desorption/ionization time‐of‐flight mass spectrometry imaging (MALDI‐TOF MSI) of a longitudinal section of a vegetative winter bud of Picea abies. (a) The bud section (approx. 20‐μm thick) was fixed on the MALDI target using a conductive double‐sided adhesive tape, covered with 2,5‐dihydroxybenzoic acid matrix and rastered (laser spot size was set to 35 μm) at a lateral resolution of 100 μm. In the positive‐ion mode three prominent signals were observed at m/z 381, m/z 543 and m/z 705 in the lower region of the primordium. The corresponding ion images (in false colors cyan, red and yellow; relative signal intensities were in the range from 0 to 33%) are depicted in Panels b–d. (c) Average MALDI‐TOF mass spectrum in the range of m/z 350 to m/z 750 found in the defined region of interest (ROI as shown in the insert of Panel D). The signals were identified as potassiated quasimolecular ions ([M + K]^+^) of a disaccharide ([C_12_H_22_O_11_ + K]^+^; calc. m/z 381.0794), a trisaccharide ([C_18_H_32_O_16_ + K]^+^; calc. m/z 543.1322) and a tetrasaccharide ([C_24_H_42_O_21_ + K]^+^; calc. m/z 705.1850)

Moreover, 2D chemical mapping of longitudinal slices of P. abies buds utilizing MALDI‐TOF MSI clearly revealed the presence of oligosaccharides to be exclusively present in the lower region of the bud primordium, separated from the crown tissue and the apex of the primordium (Figure 7). According to the average spectrum depicted in (e) of Figure 7, the ratio of concentrations of di, tri and tetrasaccharides could be estimated to be 100:100:30. Three prominent signals in the positive‐ion mode at m/z 381, m/z 543 and m/z 705 were found to be related to potassiated quasimolecular ions ([M + K]^+^) of a disaccharide ([C_12_H_22_O_11_ + K]^+^; calc. m/z 381.0794), a trisaccharide ([C_18_H_32_O_16_ + K]^+^; calc. m/z 543.1322), and a tetrasaccharide ([C_24_H_42_O_21_ + K]^+^; calc. m/z 705.1850).

## 4 DISCUSSION

### 4.1 Freezing pattern and structural requirements for supercooling

The remarkable supercooling ability of bud primordia of P. abies is facilitated by extrinsic and intrinsic ice barriers and the absence of active ice nucleators. After mechanical destruction of the bud scales or vacuum infiltration with water, supercooling ability was completely lost. Isolated primordia of larch (Larix kaempferi) were also unable to supercool (Endoh, Kuwabara, Arakawa, & Fujikawa, 2014). This suggests that the complex covering by water‐proof densely packed bud scales is essential for protection against extrinsic ice nucleation. Similarly, intact bud scales appeared to be indispensable for supercooling of flower buds of P. persica (Quamme et al., 1995). When air cushions were artificially filled with water, the primordium froze instantaneously at HTE. Evidently, the primordial epidermal tissue is not able to prevent ice penetration, which is in contrast to fully differentiated epidermal tissues of other species (Hacker & Neuner, 2008; Neuner & Hacker, 2012; Wisniewski, Lindow, & Ashworth, 1997).

Ice nucleated in the shoot around −10.6 °C, which corresponds to earlier findings (Buchner & Neuner, 2009; Pukacki, 1987). Then ice was monitored to spread rapidly via the xylem throughout the whole shoot, further into intracellulars of the bark and into the base of the bud scales. Intrinsic ice nucleation of the primordium was apparently inhibited by a bowl‐shaped tissue, which confines the primordium from the shoot, and apparently acts as an ice barrier. Anatomical characteristics of this tissue are similar to those of reproductive overwintering buds of angiosperms (e.g., Ashworth, 1984; Jones et al., 2000; Quamme et al., 1995), that is, small tightly packed cells with thick walls that are devoid of vacuoles and intercellular spaces.

Under the experimental conditions in all three buds, ice nucleated spontaneously inside the primordium (between −18.2 °C and −21.4 °C) and did not enter via the crown tissue. Evidently below a certain temperature threshold, ice nucleators inside the primordium become active and trigger intracellular ice nucleation. Similar observations were reported for supercooling reproductive organs of other woody plants (Kuprian et al., 2016; Kuprian, Briceño, Wagner, & Neuner, 2014; Neuner & Kuprian, 2014; Wisniewski, Gusta, & Neuner, 2014). This freezing pattern is in contrast to that in supercooling xylem parenchyma cells (XPCs; Neuner, Xu, & Hacker, 2010). XPCs do not freeze as complete rays, but rather in single cells or small groups of cells over a long time period. This suggests the existence of an ice barrier between XPCs (Hong & Sucoff, 1980; Neuner et al., 2010). These types of barriers are evidently absent between cells of the primodial tissue of P. abies.

### 4.2 Freeze dehydration and extraorgan freezing

Freeze dehydration of primordia appeared to have two major effects, that is, prevention of internal ice formation and enhancement of supercooling (Ide, Price, Arata, & Ishikawa, 1998). In winter, the water potential of unfrozen primordia of P. abies was at mean −3.5 ± 0.3 MPa (Figure 4). This corresponds well with osmotic potentials of bud primordia (−3.0 MPa) of Malus domestica (Pramsohler & Neuner, 2013). In contrast, to P. abies, however, buds of M. domestica supercool but do not freeze intracellularly upon frost damage.

Adjustments of water relations during winter have generally been considered to play a key role in frost survival of tissues (Margesin, Neuner, & Storey, 2007; Zwiazek et al., 2001). Reduced water potentials during winter in buds may be a general feature and originate from dehydration but also from accumulation of osmotically active substances, such as sugars detected by MALDI‐TOF.

Supercooled primordia of P. abies get freeze dehydrated, and dehydration is temperature dependent (Figure 5). During controlled freezing water, migration out of the primordium was 1.5‐fold higher in January versus April. This may indicate that water migration from the primordium to extraorgan ice masses is in winter more effectively performed across the barrier. The freeze dehydration rate could either be influenced by the water potential gradient or by changes of the permeability of the barrier.

A comparable extent of freeze dehydration has been reported for buds of M. domestica (Pramsohler & Neuner, 2013) and florets of *Rhododendron sp*. (Ishikawa & Sakai, 1981). Exposure of primordial cells to freeze dehydration may require similar physiological provisions as frost tolerant cells that freeze extracellularly. As freeze dehydration is also observed in bud primordia that do not supercool, freeze dehydration may not be a unique feature of supercooling primordia.

Water removed out of the primordium of P. abies freezes extraorgan below the crown. These ice masses form a large cavity and destroy the pith parenchyma tissue by mechanical disruption. Other conifers with supercooling bud primordia also seem to form such cavities (Lewis & Dowding, 1924). Freezing of this removed water was not detectable by IDTA, as the efflux of water might be too slow for detection.

### 4.3 Peculiar chemical features of the ice barrier tissue and the primordium

Within and between the cell walls of the bowl‐shaped ice barrier tissue, a high content of pectins was detected. The functional role of pectins has been demonstrated in peach buds where a degradation of pectins and/or chelating of Ca^2+^ resulted in the elimination of supercooling (Wisniewski & Davis, 1995). Pectins reduce the pore size diameter in cell walls (Albersheim et al., 2010). This could make cell walls impermeable for ice because water in small pores freezes at successively lower temperatures (Ashworth & Abeles, 1984). A small pore size diameter in cell walls is a feature of supercooling conifers because cell wall pores of these species were found to be smaller than in species that do not supercool (Zwiazek et al., 2001).

Cell walls of the ice barrier were not lignified or suberized, which, in contrast, appeared to contribute to supercooling of *Rhododendron* bud primordia (Chalker‐Scott, 1992); however, a peculiar distribution pattern for proteins was detected (Figure 6b). Proteins related to cold hardiness can occur in the cytosol or are secreted into the apoplast and have osmoregulatory and cryoprotective function or even antifreeze activity (Margesin et al., 2007). Apoplastic proteins reportedly have cell wall modifying functions are pathogenesis related or when bound to cell walls or cell membranes have antifreeze capacity (Griffith et al., 1992). In P. abies, they may also aid in directing water towards ice masses in the shoot. Proteins can additionally have Ca^2+^‐binding activity (Puhakainen et al., 2004), which could contribute to an interaction with pectins.

Supercooling capacity of P. abies bud primordia increases with season (from summer to winter) by 19.3 K (−5.3 °C to −24.6 °C; Kuprian et al., 2017). The underlying biochemical changes in the tissues of P. abies buds are under investigation (Oberhammer, 2016). Preliminary results suggest that the molecular distribution patterns in bud primordia in summer and winter are quite similar with starch and amino acids forming prominent components and distinct difference between the primordial tissue, the ice barrier and the stem tissue below by this corroborating the results of Raman spectroscopy.

In primordial cells close to the crown tissue, an accumulation of amorphous starch and of di, tri and tetrasaccharides was detected. Accumulation of sugars such as glucose, fructose, sucrose, and raffinose during cold acclimation of plants is well documented (Kawamura & Uemura, 2014). In addition, the accumulation of raffinose and fructose‐based oligosaccharides has been correlated to increased freezing tolerance (Kawamura & Uemura, 2014; Livingston et al., 2009). For needles of P. abies, frost hardening and dehardening correlated with an increase and decrease of sugar levels, respectively (Aronsson et al., 1976; DeHayes, 1992; Ogren et al., 1997).

Carbohydrates have long been recognized as an important contributor to freezing tolerance. Proposed mechanisms involving various forms of sugars are freezing point depression of specific tissues, prevention of cell plasmolysis as water migrates from cells during freezing, dilution of toxic compounds as cell contents are concentrated during freeze dehydration, prevention of adhesive stress as apoplastic ice approaches cells walls and membranes, alteration of glass transition temperatures during dehydration, and membrane stabilization by fructans (Green, 1983; Hincha et al., 2007; Levitt, 1959; Livingston, Olien, & Freed, 1989; Olien & Smith, 1977; Santarius, 1973; Steponkus & Lanphear, 1968; Sugiyama & Shimura, 1967; Trunova, 1965; Wolfe & Bryant, 1999). It is probable that some form of each of these mechanisms is operative in varying tissues at any given time during freezing; this makes an accurate understanding of freezing tolerance in plants a daunting task indeed.

Our results not only document peculiar cell‐specific patterns of sugar concentrations in supercooling bud primordia of P. abies but also a highly diverse layer of cells in terms of molecular compounds within the crown tissue and adjacent primordial cells. This underscores the need for a thorough, tissue and/or cell‐specific analysis of tissues that contribute to survival if freezing tolerance in plants is to be accurately understood.

## Supporting information

Video S1. 3D reconstruction of a bud of Picea abies, which was frozen to ‐40 °C for 24 h, subsequently freeze‐fixed, and sectioned at 15 microns, resulting in 341 individual sections. At the beginning of the video, the whole bud and shoot can be seen (seconds 10 to 16), then the bud scales and the bark tissue become transparent (starting from second 16 on). This reveales the leave primordia, which surround the shoot primordium. The resin ducts are colored yellow (second 20 to 35), while the outer vascular tissues and bud scales are transparent. After fading of the resin ducts, the crown tissue is highlighted in red color (second 36 to 41), followed by the ice voids, which are colored blue (second 42 to 68).

Video S2. Sequence of the 341 individual images of cross sections of a whole bud of P. abies starting from the tip of the bud scales (section no. 1) to the apex of the primordium (section no. 38) and the beginning of the shoot primordium (section no. 52). The whole primordium extends from section no. 38 to no. 166. The bowl‐like ice barrier starts to get visible at section no. 155, from section no. 167 to no. 181 the crown tissue (red) can clearly be seen. The ice voids (blue) and the resin ducts (yellow) were found in all sections starting from no. 175 and no. 153 onwards, respectively.
